# Zoledronic acid overcomes chemoresistance and immunosuppression of malignant mesothelioma

**DOI:** 10.18632/oncotarget.2731

**Published:** 2014-12-31

**Authors:** Iris Chiara Salaroglio, Ivana Campia, Joanna Kopecka, Elena Gazzano, Orecchia Sara, Dario Ghigo, Chiara Riganti

**Affiliations:** ^1^ Department of Oncology, University of Torino, Italy; ^2^ S.C. Anatomia Patologica, Azienda Ospedaliera S.S. Antonio e Biagio, Alessandria, Italy

**Keywords:** malignant mesothelioma, chemoresistance, immunosuppression, indoleamine 1,2 dioxygenase, zoledronic acid

## Abstract

The human malignant mesothelioma (HMM) is characterized by a chemoresistant and immunosuppressive phenotype. An effective strategy to restore chemosensitivity and immune reactivity against HMM is lacking. We investigated whether the use of zoledronic acid is an effective chemo-immunosensitizing strategy.

We compared primary HMM samples with non-transformed mesothelial cells.

HMM cells had higher rate of cholesterol and isoprenoid synthesis, constitutive activation of Ras/extracellular signal-regulated kinase1/2 (ERK1/2)/hypoxia inducible factor-1α (HIF-1α) pathway and up-regulation of the drug efflux transporter P-glycoprotein (Pgp). By decreasing the isoprenoid supply, zoledronic acid down-regulated the Ras/ERK1/2/HIF-1α/Pgp axis and chemosensitized the HMM cells to Pgp substrates. The HMM cells also produced higher amounts of kynurenine, decreased the proliferation of T-lymphocytes and expanded the number of T-regulatory (Treg) cells. Kynurenine synthesis was due to the transcription of the indoleamine 1,2 dioxygenase (IDO) enzyme, consequent to the activation of the signal transducer and activator of transcription-3 (STAT3). By reducing the activity of the Ras/ERK1/2/STAT3/IDO axis, zoledronic acid lowered the kyurenine synthesis and the expansion of Treg cells, and increased the proliferation of T-lymphocytes.

Thanks to its ability to decrease Ras/ERK1/2 activity, which is responsible for both Pgp-mediated chemoresistance and IDO-mediated immunosuppression, zoledronic acid is an effective chemo-immunosensitizing agent in HMM cells.

## INTRODUCTION

Human malignant mesothelioma (HMM) is an asbestos-related tumor with a progressively increasing incidence in the next 10 years. The current therapy is based on pleuro-pneumonectomy, neo-adjuvant and/or adjuvant chemotherapy, radiotherapy [1]. The prognosis is poor and the overall survival is less than 1 year. Small phase I/II trials using targeted therapy - e.g. inhibitors of vascular endothelial growth factor receptor, phosphatidylinositol 3-kinase/mammalian target of rapamycin (PI3K/mTOR), histone deacetylase and proteasome [2–4] -, employing gene therapy [5] or immunotherapy [3, 6, 7], are ongoing. These approaches however do not offer significant advantages in terms of patients' outcome, and chemotherapy is still the most common therapeutic approach. The first-line chemotherapy is based on platinum salts and antifolates (such as pemetrexed or ralitrexed) [3]; multiple combinations of drugs, including mitomycin C, Vinca alkaloids, gemcitabine, anthracyclines, irinotecan [2], have been experimented as second-line treatments, with poor success.

The main reasons of the chemotherapy failure include the scanty drug delivery within pleural tissue and the chemoresistance, which occurs in up to 60% of the HMM patients at the diagnosis [8]. The activation of PI3K/Akt/mTOR [9, 10], osteopontin/Akt [11], macrophage colony stimulating factor-1 receptor/Akt [12], Mcl-1/Bcl-x_L_ [13], senescence-associated secretory phenotype/signal transducer and activator of transcription-3 (STAT3) [14] pathways, and the presence of the ATP binding cassette (ABC) transporters P-glycoprotein (Pgp), multidrug resistance related proteins (MRPs) and breast cancer resistance protein (BCRP) [10, 15–17], have been correlated with HMM chemoresistance. Pgp has the broadest spectrum of substrates, which include anthracyclines, taxanes, Vinca alkaloids, epipodophyllotoxins, topotecan, methotrexate, imatinib, dasatinib, lapatinib, gefitinib, sorafenib, erlotinib [18].

The second issue that makes HMM hard to eradicate is the tumor-induced immunosuppression [7], mainly due to the release of specific cytokines increasing the percentage of immunosuppressive regulatory T-cells (Tregs) [19], myeloid-derived suppressor cells, type 2-tumor associated macrophages (TAMs), which suppress the proliferation and effector functions of T-lymphocytes [7].

One of the strongest mediators of tumor-induced immunosuppression is kynurenine, a tryptophan catabolite produced by indoleamine 1,2 dioxygenase (IDO) [20]. The role of kynurenine in HMM has not yet been investigated.

The “ideal” therapy of HMM should improve the efficacy of chemotherapy and recover the immunosuppression induced by the HMM cells themselves.

Recently we demonstrated that the aminobisphosphonate zoledronic acid overcomes the resistance to doxorubicin by reducing the activity of Ras/extracellular signal-regulated kinase1/2 (ERK1/2)/hypoxia inducible factor-1α (HIF-1α)/Pgp axis, and restores a proper recognition by the host immune system in chemo-immunoresistant solid tumors [21]. Zoledronic acid is known to reduce HMM growth [22], induce apoptosis [23], up-regulate p53 [24], impair the polarization of TAMs towards type 2-phenotype, reduce the synthesis of immunosuppressive cytokines [25].

In this work we investigated whether the use of zoledronic acid is an effective chemo-immunosensitizing strategy in primary HMMs, thus fulfilling two requirements of the “ideal” therapy of HMM.

## RESULTS

### Zoledronic acid chemosensitizes mesothelioma cells by down-regulating the Ras/ERK1/2/HIF-1α/Pgp axis

Samples from ten HMM patients, whose demographic and histological features are reported in Table 1, were analyzed for the sensitivity to different chemotherapeutic drugs, unrelated for chemical structure and activity: irrespectively of the patients' clinical and histological features, the half maximal inhibitory concentration (IC50) for each drug (Table 2) and the resistance factor (Rf; Table 3) were significantly higher than in non-transformed human mesothelial cells (HMC).

**Table 1:** Demographic and histological features of HMM patients

UPN	Age	Sex	Histotype	Asbestos exposure	SV40 presence
1	79	M	Epithelioid	Unlikely	Negative
2	60	M	Biphasic	Professional	Negative
3	68	M	Epithelioid	Professional	Negative
4	64	M	Epithelioid	Not determined	Negative
5	70	M	Epithelioid	Professional	Negative
6	80	F	Sarcomatous	Environmental	Negative
7	89	F	Sarcomatous	Not determined	Negative
8	72	M	Biphasic	Not determined	Negative
9	62	M	Epithelioid	Professional	Positive
10	66	F	Sarcomatous	Not determined	Negative

UPN: unknown patient number

**Table 2:** IC50 (μmol/L) of different chemotherapeutic drugs in HMC and HMM cells

Sample	DOX	VBL	ETO	Pt	GEM	PMX	MXR
HMC	4.3 ± 0.12	5.5 ± 0.02	52.00 ± 6.32	9.09 ± 1.39	4.46 ± 0.82	0.89 ± 0.05	17.87 ± 1.57
UPN 1	30.05 ± 1.32	36.32 ± 0.13	169.27 ± 12.33	49.01 ± 5.21	28.06 ± 2.54	5.14 ± 0.89	90.97 ± 4.21
UPN 2	30.05 ± 2.38	39.45 ± 0.25	204.79 ± 12.41	57.47 ± 1.23	38.42 ± 2.36	6.00 ± 1.01	111.19 ± 9.56
UPN 3	31.57 ± 5.26	47.66 ± 0.51	247.58 ± 14.58	99.99 ± 4.36	50.97 ± 4.15	9.06 ± 1.58	123.55 ± 4.2
UPN 4	25.98 ± 2.56	33.68 ± 0.24	174.61 ± 9.63	53.19 ± 5.23	25.75 ± 4.23	7.22 ± 0.57	105.34 ± 8.21
UPN 5	59.38 ± 5.69	44.43 ± 0.16	230.39 ± 12.14	108.68 ± 4.23	50.97 ± 5.36	7.33 ± 1.02	238.27 ± 14.85
UPN 6	22.88 ± 4.23	33.32 ± 0.24	172.79 ± 5.23	58.82 ± 2.36	28.38 ± 1.28	5.42 ± 0.47	115.03 ± 11.03
UPN 7	28.02 ± 2.59	33.19 ± 0.16	165.88 ± 6.32	52.63 ± 4.51	26.86 ± 1.95	5.80 ± 0.12	149.36 ± 8.25
UPN 8	27.11 ± 1.06	33.51 ± 0.12	182.29 ± 11.04	54.94 ± 7.23	28.71 ± 1.87	5.60 ± 0.43	108.77 ± 5.23
UPN 9	28.02 ± 0.28	33.16 ± 0.17	164.24 ± 12.54	52.63 ± 1.45	26.86 ± 2.54	5.80 ± 0.62	181.95 ± 14.5
UPN 10	47.96 ± 8.72	44.76 ± 0.41	247.58 ± 11.32	68.49 ± 5.28	44.60 ± 1.23	10.60 ± 0.74	181.99 ± 5.48

HMC: human mesothelial cells; HMM: human malignant mesothelioma; UPN: unknown patient number; DOX: doxorubicin; VBL: vinblastine; ETO: etoposide; Pt: cisplatin; GEM: gemcitabine; PMX: pemetrexed; MXR: mitoxantrone. Significance for each drug, HMM cells versus HMC: *p* < 0.01.

**Table 3:** Resistance factor (Rf) for different chemotherapeutic drugs in HMM cells

	DOX	VBL	ETO	Pt	GEM	PMX	MXR
UPN 1	6.99	5.92	5.11	5.39	6.29	5.77	5.09
UPN 2	6.99	7.16	6.08	6.32	8.62	6.75	6.22
UPN 3	7.34	8.66	6.86	11	11.43	10.18	6.91
UPN 4	6.04	6.11	4.62	5.85	5.77	8.12	5.89
UPN 5	13.81	8.06	7.62	11.96	11.43	8.24	13.33
UPN 6	5.32	6.04	5.39	6.47	6.36	6.09	6.44
UPN 7	6.52	5.8	5.22	5.79	6.02	6.51	8.36
UPN 8	6.3	6.37	5.27	6.04	6.44	6.29	6.09
UPN 9	6.52	5.74	5.22	5.79	6.02	6.51	10.18
UPN 10	11.15	8.66	7.62	7.53	10	11.91	10.18

Resistance factor (Rf) was calculated by dividing the IC50 of each drug in human malignant mesothelioma HMM cells for the corresponding IC50 in human mesothelial cells HMC (see Table 2).

UPN: unknown patient number; DOX: doxorubicin; VBL: vinblastine; ETO: etoposide; Pt: cisplatin; GEM: gemcitabine; PMX: pemetrexed; MXR: mitoxantrone.

Significance for each drug. HMM cells versus HMC: *p* < 0.001.

**Figure 1:**
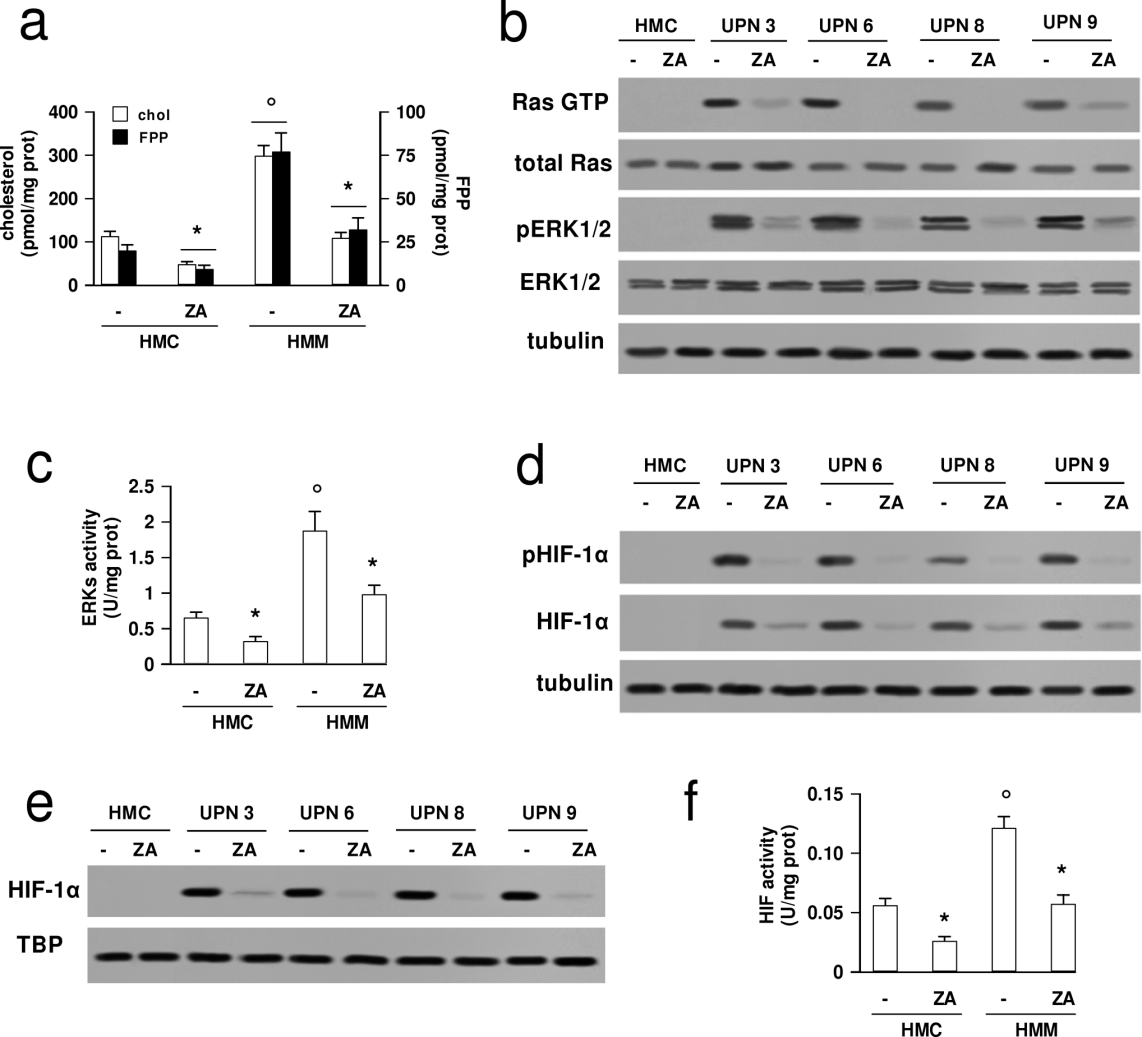
Zoledronic acid down-regulates Ras/ERK1/2/HIF-1α axis in mesothelioma cells HMC and HMM cells (UPN, unknown patient number) were incubated in fresh medium (−) or in the presence of 1 μmol/L zoledronic acid (ZA) for 48 h, then subjected to the following investigations. **(a)** The cells were radiolabeled during the last 24 h with [^3^H]acetate, then the *de novo* synthesis of cholesterol (open bars) or FPP (solid bars) was measured as described in Materials and methods. Data are presented as means ± SD (*n* = 3). Vs untreated (−) cells: *p < 0.01; HMM cells vs HMC: °*p* < 0.005. **(b)** The cells were lysed and subjected to the Western blot analysis for Ras-GTP (as index of active Ras), total Ras, phospho(Thr202/Tyr204, Thr185/Tyr187)-ERK1/2, total ERK1/2. The β-tubulin expression was used as control of equal protein loading. The figure is representative of 3 experiments with similar results. **(c)** The ERK activity was measured in cell lysates by a specific ELISA kit. Data are presented as means ± SD (*n* = 3). Vs untreated (−) cells: *p < 0.01; HMM cells vs HMC: °*p* < 0.01. **(d)** Cells were lysed and subjected to the Western blot analysis for phospho(Ser)-HIF-1α and total HIF-1α. The β-tubulin expression was used as control of equal protein loading. The figure is representative of 3 experiments with similar results. **(e)** The amount of HIF-1α was measured in nuclear extracts by Western blotting. The TBP expression was used as control of equal protein loading. The figure is representative of 3 experiments with similar results. **(f)** HIF-1α activity was measured in nuclear extracts by a specific ELISA kit. For each set of experiments, a competition assay (using 20 pmol of the wild type oligonucleotide with nuclear extracts from UPN1 cells grown at 3% O_2_ for 24 h) was included. In hypoxic conditions, the activity of HIF-1α was 58.32 ± 7.97 U/mg proteins; in the competition assay, the corresponding HIF-1α activity was reduced to 5.09 ± 0.56 U/mg proteins (*n* = 3; *p* < 0.001). Data are presented as means ± SD (*n* = 3). Vs untreated (−) cells: *p < 0.01; HMM cells vs HMC: °*p* < 0.001.

All HMM samples expressed Pgp, MRP1 and MRP3, and showed variable amounts of MRP4, MRP5, BCRP; such ABC transporters were undetectable in HMC (Supplementary Figure 1). In the subsequent experiments, all the 10 samples (indicated as HMM cells) were investigated, but in blotting experiments, for sake of simplicity, we show four out of the ten samples, including one sarcomatous, two epithelioid (one SV-40-negative and one SV-40-positive) and one biphasic histotypes. However, no significant differences in the parameters examined were observed as a function of the histotype (see below). Compared to the HMC, the HMM cells had higher cholesterol and farnesyl pyrophosphate (FPP) synthesis (Figure 1a), and showed higher levels of GTP-bound Ras (Figure 1b), phosphorylation (Figure 1b) and activity (Figure 1c) of the Ras downstream effectors ERK1/2. In HMM cells, but not in HMC, the transcription factor HIF-1α, a known inducer of Pgp and a target of Ras/ERK1/2 axis [21], was phosphorylated on serine (Figure 1d), translocated in the nucleus (Figure 1e) and bound to the target DNA (Figure 1f). By inhibiting the FPP synthase [26] and reducing the FPP supply (Figure 1a) necessary for Ras, zoledronic acid significantly lowered the activation of Ras, ERK1/2 and HIF-1α (Figures 1b–f).

**Figure 2:**
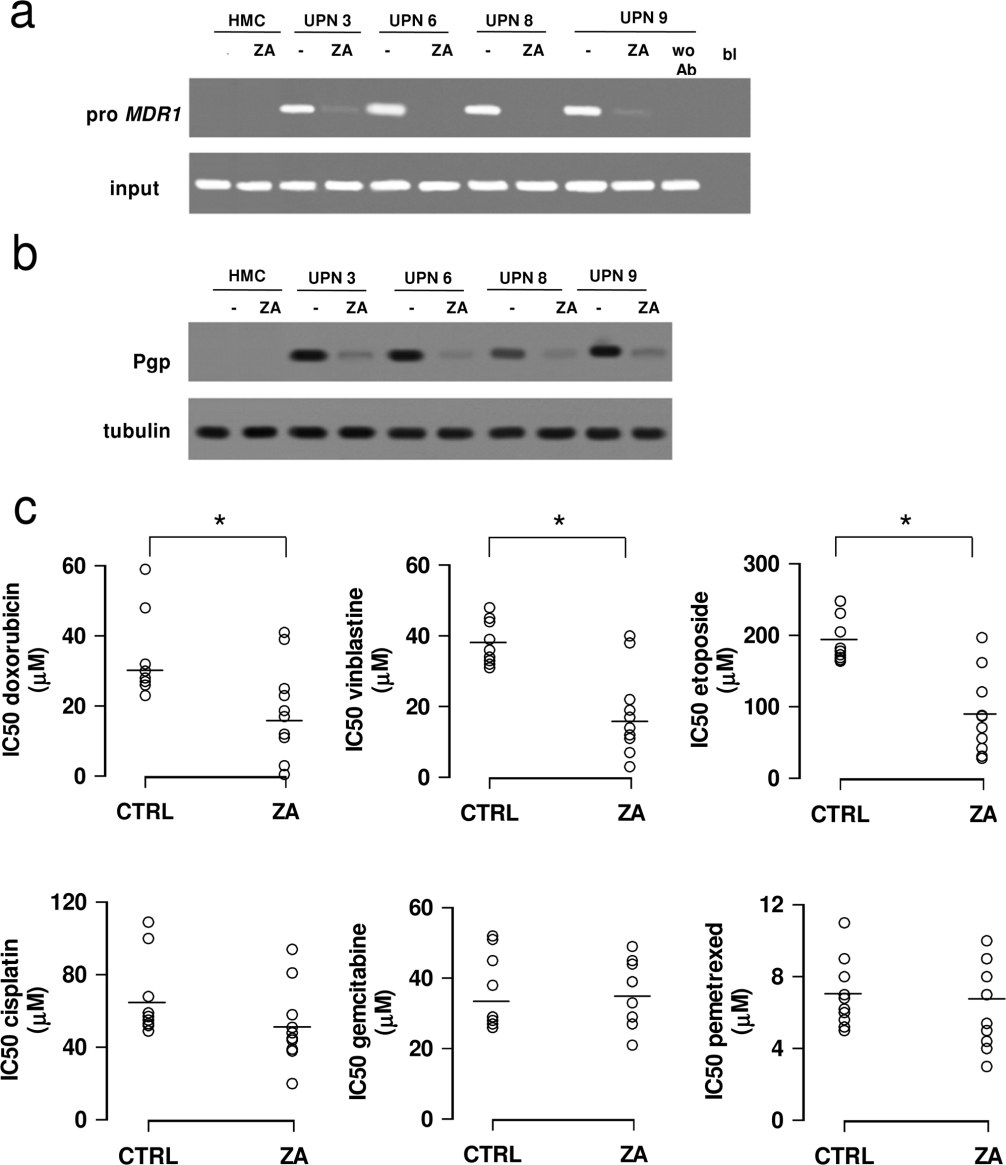
Zoledronic acid chemosensitizes mesothelioma cells to Pgp substrates HMC and HMM cells (UPN, unknown patient number) were incubated in fresh medium (−) or in the presence of 1 μmol/L zoledronic acid (ZA) for 48 h, then subjected to the following investigations. **(a)** ChIP of HIF-1α on *MDR1* promoter (pro MDR1). wo Ab: samples precipitated without anti-HIF-1α antibody; bl: blank; input: genomic DNA. The figure is representative of 3 experiments with similar results. **(b)** Cells were lysed and subjected to the Western blot analysis for Pgp expression. The β-tubulin expression was used as control of equal protein loading. The figure is representative of 3 experiments with similar results. **(c)** IC50 for doxorubicin, vinblastine, etoposide, cisplatin, gemcitabine and pemetrexed in HMM cells. The cells were incubated with increasing concentrations of each drug (1-10-100 pmol/L, 1-10-100 nmol/L, 1-10-100 μmol/L, 1 mmol/L) for 48 h, in the absence (CTRL) or in the presence of 1 μmol/L ZA. Cell viability was assessed with neutral red staining, as detailed under Materials and methods. Data are presented as means ± SD (*n* = 4). Doxorubicin-, vinblastine-, etoposide-treated cells vs CTRL cells: *p < 0.02.

HIF-1α was constitutively bound to the promoter of *MDR1* gene, which encodes for Pgp, in HMM cells (Figure 2a) that – differently from HMC – were characterized by constitutively detectable levels of Pgp protein (Figure 2b). Zoledronic acid reduced the binding of HIF-1α to the *MDR1* promoter (Figure 2a) and the expression of Pgp (Figure 2b). Consequently, it lowered the IC50 of chemotherapeutic drugs that are substrates of Pgp (Supplementary Table 1), such as doxorubicin, vinblastine and etoposide (Figure 2c). By contrast, zoledronic acid did not affect the IC50 of cisplatin, gemcitabine and pemetrexed (Figure 2c) that are not effluxed by Pgp (Supplementary Table 1).

The mean IC50 of zoledronic acid alone in HMM samples was 96.3 ± 8.7 μmol/L, nearly 100-fold higher than the concentration (1 μmol/L) used in all our experiments. Such difference led to exclude that zoledronic acid exerts a cytotoxic effect in HMM cells at the concentration used in the present work.

The combination index (CI) of 1 μmol/L zoledronic acid and different concentrations (from 1 pmol/L to 1 mmol/L) of chemotherapeutic drugs is reported in the Supplementary Table 2 and in the Supplementary Figure 2: whereas for most concentrations of doxorubicin, vinblastine and etoposide the effect of zoledronic acid was synergistic, for most concentrations of cisplatin, gemcitabine and pemetrexed the effect was additive (Supplementary Figure 2). Focusing on the concentrations around the IC50 of each chemotherapeutic drug in the presence of zoledronic acid, we found that the aminobisphosphonate produced clear synergistic effects in the case of doxorubicin, vinblastine and etoposide, additive effects or even slightly antagonistic effects in the case of cisplatin, gemcitabine and pemetrexed (Supplementary Table 2).

As shown in the Supplementary Table 1, ABC transporters other than Pgp mediate the resistance towards cisplatin, gemcitabine and pemetrexed. Differently from what observed on Pgp levels, zoledronic acid did not reduce the expression of MRP1, MRP2, MRP4 and MRP5 (Supplementary Figure 3a), the transporters involved in the efflux of cisplatin, gemcitabine and pemetrexed (Supplementary Table 1). The amount of cisplatin, gemcitabine and pemetrexed retained within HMM cells was sufficient to exert the typical anti-tumor actions of these drugs. Cisplatin induced DNA damage (Supplementary Figure 3b). Gemcitabine impaired the cell cycle progression by increasing the percentage of apoptotic cells and of cells blocked in S-phase, thus inducing a mitotic catastrophe (Supplementary Figure 3c). Pemetrexed inhibited the target enzyme dihydrofolate reductase (DHFR; Supplementary Figure 3d). As to all these parameters, however, zoledronic acid did not enhance the anti-tumor effects induced by the chemotherapeutic drugs (Supplementary Figure 3b–d).

### Zoledronic acid immunosensitizes mesothelioma cells by lowering the expression and activity of IDO in a Ras/ERK1/2/STAT3-dependent way

**Figure 3:**
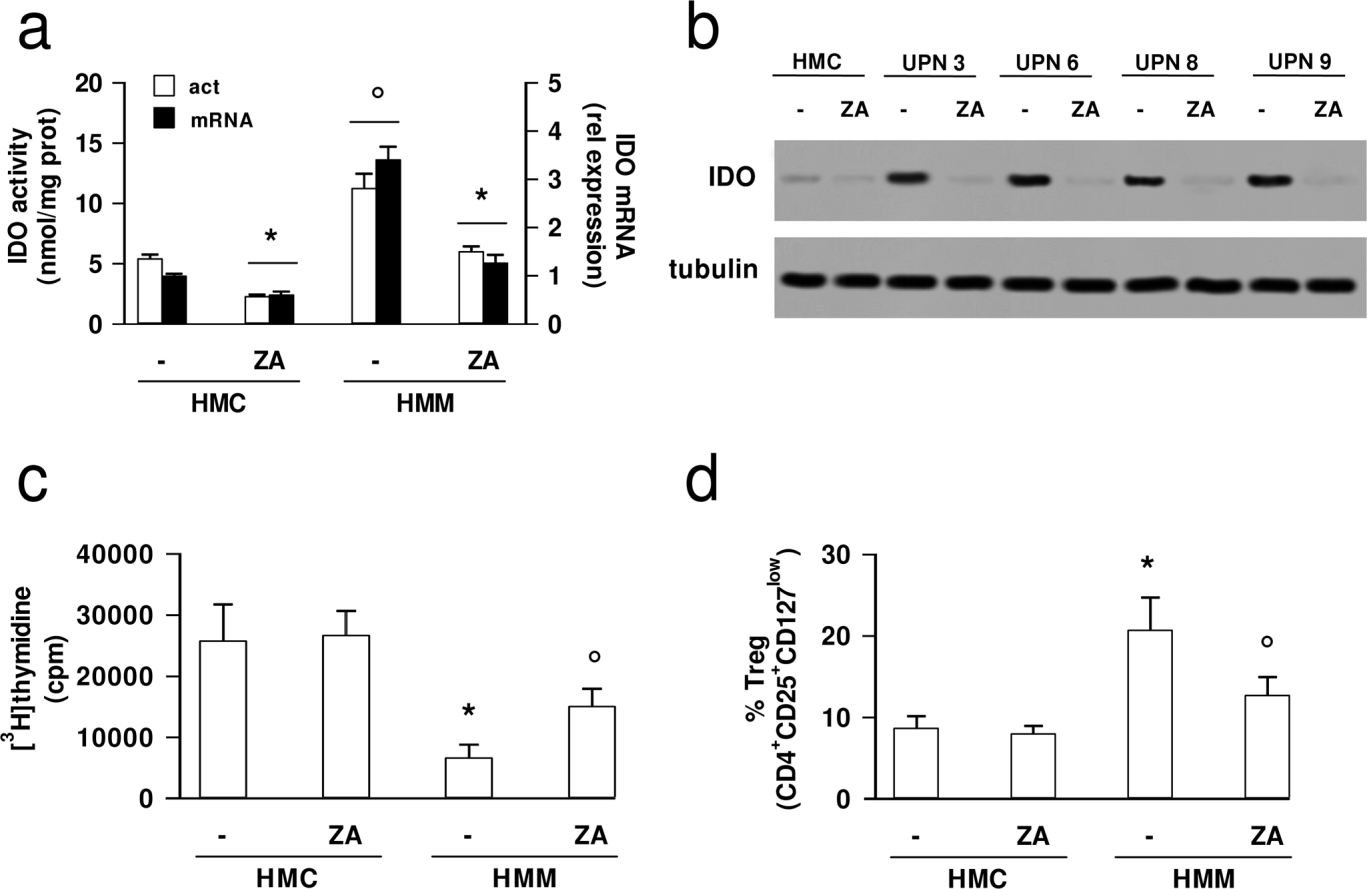
Zoledronic acid down-regulates IDO expression in mesothelioma cells and inhibits the tumor-induced immunosuppression HMC and HMM cells (UPN, unknown patient number) were incubated in fresh medium (−) or in the presence of 1 μmol/L zoledronic acid (ZA) for 48 h, then subjected to the following investigations. **(a)** The kynurenine level in cells culture supernatant, taken as an index of IDO enzymatic activity, was measured fluorimetrically (open bars, act); the expression levels of IDO mRNA were measured by qRT-PCR (solid bars, mRNA). Data are presented as means ± SD (*n* = 4). Vs untreated (−) cells: *p < 0.01; HMM cells vs HMC: °*p* < 0.002. **(b)** The cells were lysed and subjected to Western blot analysis for IDO expression. The β-tubulin expression was used as control of equal protein loading. The figure is representative of 3 experiments with similar results. **(c)** The proliferation of activated T-lymphocytes collected from PBMC after a 72 h co-incubation with HMC and HMM cells, grown in fresh medium (−) or in medium containing 1 μmol/L ZA, was measured with the [^3^H]thymidine assay. As positive control of proliferation, the PBMC were treated with the anti-CD3 and anti-CD28 antibodies, in the absence of cells; as negative control, the PBMC were grown in RPMI-1640 medium, in the absence of anti-CD3 and anti-CD28 antibodies, and of cells. In the presence of the anti-CD3 and anti-CD28 stimulatory antibodies, the [^3^H]thymidine incorporation was 33,123 ± 3,256 cpm; in the presence of RPMI-1640 medium alone, the [^3^H]thymidine incorporation was 3,892 ± 297 cpm. The [^3^H]thymidine incorporation in HMC and HMM cells were: 480 ± 190 cpm (untreated HMC); 482 ± 44 cpm (ZA-treated HMC); 485 ± 277 cpm (untreated HMM cells); 289 ± 28 cpm (ZA-treated HMM cells). Data are presented as means ± SD (*n* = 3). HMM cells vs HMC: *p < 0.01; ZA-treated HMM cells vs untreated (−) cells: °*p* < 0.05. **(d)** The percentage of Tregs (CD4^+^CD25^+^CD127^low^) collected from PBMC, co-incubated as reported in **c**, was measured by flow cytometry. Data are presented as means ± SD (*n* = 3). HMM cells vs HMC: *p < 0.02; ZA-treated HMM cells vs untreated (−) cells: °*p* < 0.05.

Primary HMM cells exhibited higher synthesis of the IDO-derived immunosuppressive mediator kynurenine, higher levels of IDO mRNA and protein than HMC, all reduced by zoledronic acid (Figures 3a–b).

Moreover, HMC stimulated T-lymphocyte proliferation more than HMM cells, but zoledronic acid-treated HMM cells significantly increased T-cell proliferation (Figure 3c). The percentages of CD3^+^ T-cells, CD4^+^ T-helper cells, CD8^+^ T-cytotoxic cells did not differ between HMC and HMM cells, both with or without zoledronic acid (Supplementary Figure 4a–c). Interestingly, the HMM cells expanded the number of Tregs, and zoledronic acid counteracted this event (Figure 3d). The IDO inhibitor 5-Br-brassinin [27], which actually decreased the activity of IDO in HMM cells (Supplementary Figure 5a), caused a response similar to zoledronic acid (Supplementary Figure 5b–c), suggesting that high kynurenine levels were accompanied by reduced T-lymphocyte proliferation and higher Tregs expansion, whereas low kynurenine levels – induced by zoledronic acid or 5-Br-brassinin – were paralleled by an opposite scenario.

**Figure 4:**
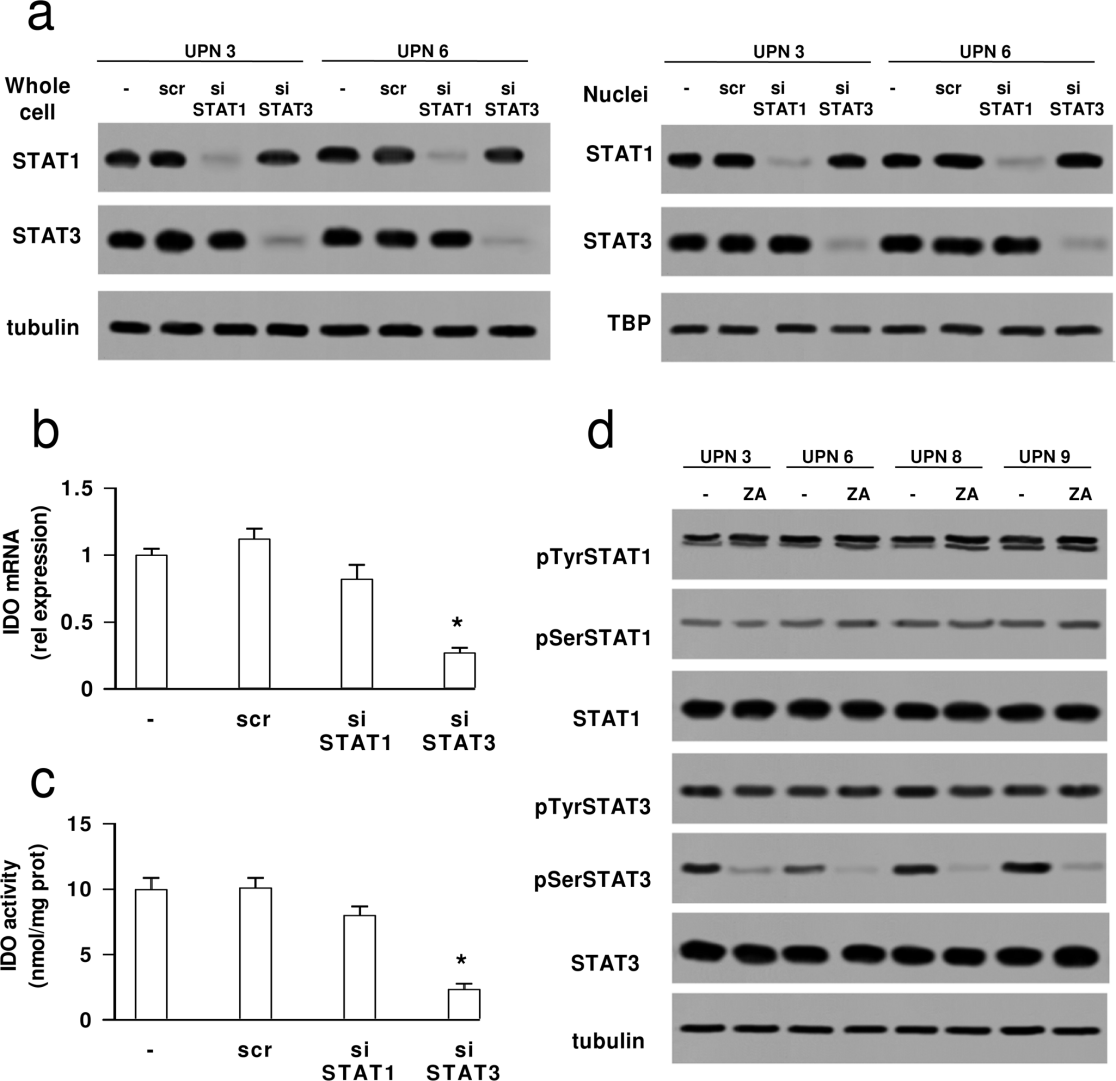
Zoledronic acid reduces the phosphorylation of STAT3, which is critical to up-regulate IDO in mesothelioma cells HMM samples (UPN: unknown patient number) were grown for 24 h in fresh medium (−), treated with a non-targeting scrambled siRNA (scr) or with a specific siRNA pool targeting STAT1 or STAT3 (siSTAT1, siSTAT3). **(a)** The expression of STAT1 and STAT3 was measured in whole cell lysates (left panel) and in nuclear extracts (right panel) by Western blotting. The β-tubulin and TBP expression were used as controls of equal protein loading in whole cell lysates and nuclear extracts, respectively. The figure is representative of 3 experiments with similar results. **(b)** The expression levels of IDO mRNA were measured by qRT-PCR. Data are presented as means ± SD (*n* = 4). Vs untreated (−) cells: *p < 0.001. **(c)** The kynurenine level in the cell culture supernatant, taken as an index of IDO enzymatic activity, was measured fluorimetrically. Data are presented as means ± SD (*n* = 4). Vs untreated (−) cells: *p < 0.001. **(d)** The cells were grown in fresh medium (−) or in the presence of 1 μmol/L zoledronic acid (ZA) for 48 h, then lysed and subjected to the Western blot analysis of phospho(Tyr701)-STAT1, phospho(Ser727)-STAT1, total STAT1, phospho(Tyr705)-STAT3, phospho(Ser727)-STAT3, total STAT3. The β-tubulin expression was used as control of equal protein loading. The figure is representative of 3 experiments with similar results.

The transcriptional activators of the *IDO* gene STAT1 and STAT3 [28, 29] were present in HMM cells and constitutively translocated in the nucleus (Figure 4a). To investigate their involvement in the transcription of *IDO*, STAT1 and STAT3 were separately silenced in two primary HMM samples (one epithelioid and one sarcomatous; Figure 4a). STAT3-, but not STAT1-silenced cells showed decreased IDO mRNA (Figure 4b) and activity (Figure 4c).

The phosphorylation of STATs on tyrosine [30] and serine [31, 32] is critical for their transcriptional activity. In HMM cells STAT1 was constitutively phosphorylated on tyrosine 701 and serine 727, STAT3 was constitutively phosphorylated on tyrosine 705 and serine 727 (Figure 4d). Zoledronic acid specifically reduced the phosphorylation of STAT3 on serine 727 (Figure 4d).

**Figure 5:**
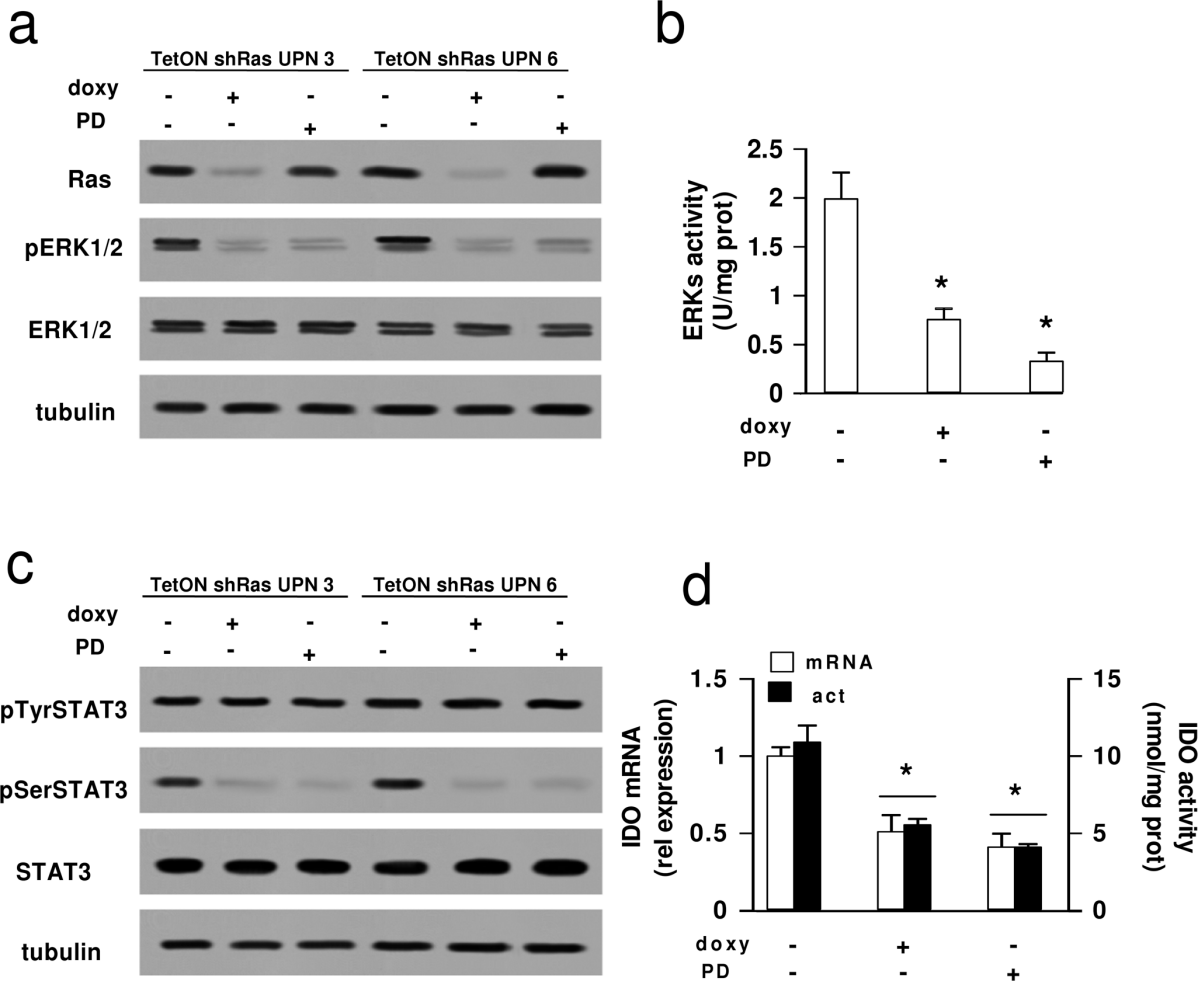
Effects of Ras/ERK1/2 inhibition on STAT3 phosphorylation and IDO expression in mesothelioma cells HMM samples (UPN: unknown patients number), treated with a TetON vector containing a shRNA for *RAS* (sh) or with the ERK1/2 inhibitor PD98059 (10 μmol/L for 24 h, PD), were cultured in medium without (−) or with (+) 1 μg/mL doxycycline (doxy). After 24 h, the following investigations were performed. **(a)** The expression of Ras, phospho(Thr202/Tyr204, Thr185/Tyr187)-ERK1/2, total ERK1/2 was measured in whole cell lysates by Western blotting. The β-tubulin expression was used as control of equal protein loading. The figure is representative of 3 experiments with similar results. **(b)** The ERK activity was measured in cell lysates using a specific ELISA kit. Data are presented as means ± SD (*n* = 3). Vs untreated (doxy -, PD -) cells: *p < 0.001. **(c)** The expression of phospho(Tyr705)-STAT3, phospho(Ser727)-STAT3, total STAT3 was assessed in whole cell lysates by Western blotting. The β-tubulin expression was used as control of equal protein loading. The figure is representative of 3 experiments with similar results. **(d)** The relative expression levels of IDO mRNA were measured by qRT-PCR (open bars, mRNA); the kynurenine level in cell culture supernatant, taken as an index of IDO enzymatic activity, was measured fluorimetrically (solid bars, act). Data are presented as means ± SD (*n* = 4). Vs untreated (doxy -, PD -) cells: *p < 0.005.

Since Ras/ERK1/2 axis is involved in the serine phosphorylation of STAT3 [33, 34], we next investigated whether the zoledronic acid's effect was mediated by the down-regulation of Ras and ERK1/2 activity. To this aim, we produced two HMM clones (one epithelioid and one sarcomatous) stably and inducibly silenced for Ras: both clones showed decreased expression of Ras and phosphorylation of ERK1/2 (Figure 5a). The same clones were also incubated with the ERK1/2 inhibitor PD98059, used as second tool to block the Ras/ERK1/2 pathway. Compared to parental cells, both Ras-silenced and PD98059-treated cells showed lower ERK activity (Figure 5b), reduced levels of phospho(Ser727)-STAT3 without changes in phospho(Tyr705)-STAT3 or total STAT3 (Figure 5c), lower IDO activity and mRNA (Figure 5d), reproducing the situation observed in HMM cells treated with zoledronic acid.

## DISCUSSION

In this work we found that the aminobisphosphonate zoledronic acid reverses chemoresistance and immunosuppression of HMM cells.

Since HMM patients vary for genetic aberrations, histological features, absence or presence of pro-oncogenic factors, we collected a series of HMM primary samples, homogeneous for the demographic features, but different for the tumor histotype and etiology. One epithelioid sample contained SV40 whole genome, which has been related to a highly aggressive phenotype [35]. All samples had a multidrug resistant phenotype and were significantly more chemoresistant than the non-transformed HMC, owing to the constitutive presence of several ABC transporters.

As it occurs in other chemoresistant cells [21], the HMM cells displayed a higher synthesis of cholesterol and isoprenoid units like FPP, and a higher activity of Ras and downstream effectors implicated in the chemoresistance [21], compared to the chemosensitive HMC. Ras/ERK1/2 axis is a critical pro-oncogenic pathway in HMM [36]. It has been reported that HMM cells silenced for ERK1/2 are more sensitive to doxorubicin and have a lower expression of Pgp [37]: our results provide an explanatory mechanism for this observation, showing that Pgp transcription is sustained by the Ras/ERK1/2/HIF-1α axis in this tumor.

Zoledronic acid, which inhibited the Ras/ERK1/2/HIF-1α/Pgp pathway, induced an appreciable chemosensitization of HMM cells. Such effect was specifically linked to the decrease of Pgp. Zoledronic acid indeed decreased the expression of Pgp, allowing Pgp substrates – such as doxorubicin, vinblastine and etoposide – to reach higher intracellular accumulation and toxicity. On the other hand, we did not observe any sensitization towards substrates of other ABC transporters, such as cisplatin, gemcitabine and pemetrexed. This can be due to the lack of down-regulation of the ABC transporters that extrude these drugs, and/or to the lack of effects on the classical cellular targets of cisplatin, gemcitabine and pemetrexed. These factors may explain the different pharmacological profile (i.e. synergistic versus additive/antagonistic effects) between substrates and non-substrates of Pgp in the presence of zoledronic acid. Although Pgp substrates are not included in the first-line therapy of HMM, most of them are used in second-line protocols [2–4]: reducing the Pgp amount is important to improve the therapeutic efficacy of either conventional chemotherapeutic drugs or new targeted drugs effluxed by this transporter [18]. The concentration of zoledronic acid that we used was not toxic and was compatible with the blood concentration observed in patients [38], making zoledronic acid potentially applicable to clinical protocols for HMM in association with chemotherapy.

Besides chemoresistance, the tumor-induced immunosuppression makes HMM an aggressive tumor. The role of the immunosuppressive metabolite kynurenine, as well as the molecular pathways up-regulating the kynurenine producing enzyme IDO, has not been investigated in HMM. We found that kynurenine production and IDO expression were significantly higher in HMM cells than in HMC, leading to hypothesize that the increase of IDO and the immunosuppressive phenotype are associated with the malignant transformation of mesothelium. The higher IDO activity was paralleled by the reduced proliferation of T-lymphocytes and by the increased expansion of the immunosuppressive Tregs subpopulation. Of note, zoledronic acid, which down-regulated IDO, restored the proliferation of T-lymphocytes and decreased the expansion of Tregs. The increased number of Tregs has been previously related to the HMM-induced immunosuppression [19, 39], but not to the production of kynurenine. These data are the premise to our in progress studies in immunocompetent animal models, aimed at investigating whether kynurenine is the actual responsible for the Tregs expansion and for the mesothelioma-induced immunosuppression *in vivo*.

Since mitogen-activated protein (MAP) kinases may activate STAT proteins [33, 34], which in turn induce *IDO* transcription [28, 29], we next investigated if in HMM cells – that exhibit a constitutively active Ras/ERK1/2 axis – IDO was induced by STAT activity and if zoledronic acid interfered with it. Both STAT1 and STAT3 were activated in untreated HMM cells, in line with previous reports [40]. The transient silencing of STAT1 and STAT3 suggested that only the latter was the transcriptional activator of *IDO* in HMM. Whereas Janus kinases-1 and -2 promote the phosphorylation of STAT3 on tyrosine, MAP kinases induce the phosphorylation on serine [33]. STAT3 is a good substrate for ERK1/2 kinases [41], which promote the transcriptional activity of STAT3 [42]. Zoledronic acid reduced the phosphorylation of STAT3 on serine 727. Of note, Ras-silenced and ERK1/2-inhibited HMM clones showed decreased levels of phospho(Ser727)-STAT3, *IDO* transcription and kynurenine synthesis. This pattern reproduced the changes elicited by zoledronic acid, suggesting that aminobisphosphonate counteracts the HMM-induced production of kynurenine by inhibiting the Ras/ERK1/2/STAT3 axis. The decrease of STAT3 activity has been exploited *in vitro* and *in vivo* as a pro-apoptotic strategy against HMM [43]. We suggest that lowering the Ras/ERK1/2/STAT3 axis can be also an effective immunosensitizing strategy.

**Figure 6:**
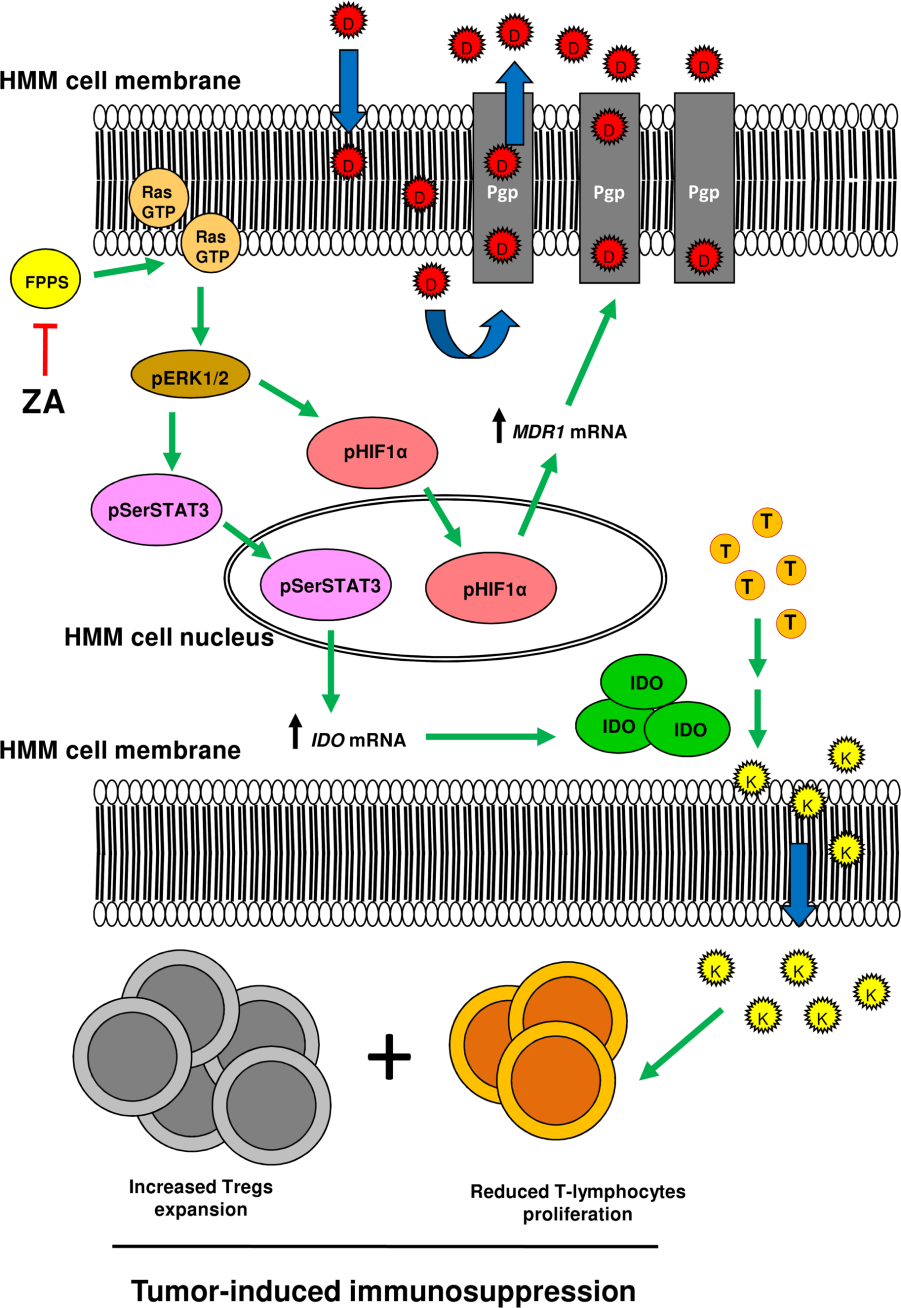
Zoledronic acid induces chemo-immunosensitization in mesothelioma cells by downregulating the Ras/ERK1/2/HIF-1α/Pgp and Ras/ERK1/2/STAT3/IDO axes HMM cells have the Ras small GTPase (Ras GTP) and the downstream effectors ERK1/2 kinases (pERK1/2) in a constitutively active form. ERK1/2 kinases phosphorylate on serine the transcription factor HIF-1α (pHIF1α), which is primed to translocate in the nucleus and to increase the transcription of the *MDR1* gene, which is translated into Pgp, an efflux transporter of chemotherapeutic drugs (D). Moreover, ERK1/2 kinases phosphorylate STAT3 (pSerSTAT3) that activates the transcription of the *IDO* gene. The increase of kynurenine (K), a product of IDO activity during the catabolism of tryptophan (T), is paralleled by the decreased T-lymphocyte proliferation and by the increased expansion of the immunosuppressive Tregs subpopulation. These events may confer to mesothelioma a chemoresistant and immunosuppressive phenotype. By inhibiting the FPP synthase (FPPS) and then reducing the supply of FPP necessary to activate Ras, zoledronic acid (ZA) inhibits both the Ras/ERK1/2/HIF-1α/Pgp and the Ras/ERK1/2/STAT3/IDO axes, thus potentially inducing chemo-immunosensitization. Green arrow: stimulation; red line: inhibition; blue arrow: transmembrane diffusion or transport.

In summary, we propose that the constitutively active Ras/ERK1/2 axis may determine both the chemoresistance and the immunosuppression observed in HMM. By phosphorylating and activating HIF-1α, this axis promotes the up-regulation of Pgp, which effluxes several chemotherapeutic drugs. By phosphorylating and activating STAT3, it favors the synthesis of kynurenine, which is paralleled by the reduced proliferation of T-lymphocytes and by the increased expansion of the pro-immunosuppressive Tregs population. Thanks to its ability to decrease isoprenoid synthesis and Ras/ERK1/2 activity, zoledronic acid reverses the chemoresistance to Pgp substrates, lowers the kynurenine synthesis and the Tregs proliferation, and restores the proliferation of T-lymphocytes co-cultured with HMM cells (Figure 6).

By overcoming the main defects of HMM therapy – i.e. the poor efficacy of chemotherapy and the tumor-induced immunosuppression – zoledronic acid may represent a promising adjuvant drug for the future treatment protocols of HMM.

## MATERIALS AND METHODS

### Chemicals

The plasticware for cell cultures was from Falcon (Becton Dickinson, Franklin Lakes, NJ). Zoledronic acid was a gift from Novartis (Basel, Switzerland). Doxorubicin, vinblastine, etoposide, cisplatin, gemcitabine, pemetrexed, mitoxantrone, PD98059 were purchased from Sigma Chemicals Co. (St. Louis, MO). The electrophoresis reagents were obtained from Bio-Rad Laboratories (Hercules, CA). The protein content of cell lysates was assessed with the BCA kit from Sigma Chemicals Co. When not otherwise specified, all the other reagents were purchased from Sigma Chemicals Co.

### Cells

The non-transformed HMC were isolated from three patients with transudative pleural fluid due to congestive heart failure, with no history of malignant disease. The mesothelial origin of the isolated cells was confirmed by the positive immunostaining with anti-cytokeratins 8 and 18 (Becton Dickinson), anti-vimentin (Dako, Glostrup, Denmark), anti-mesothelial cell (Dako) antibodies. The primary HMM cells were collected from the Biologic Bank of Malignant Mesothelioma, S.S. Antonio e Biagio Hospital, Alessandria, Italy, where the histological characterization was performed. One sample, already described in [35], was positive for SV40 virus. The samples, collected after informed written consent, were indicated with an “Unknown Patient Number” (UPN) in the manuscript. The experimental protocol was approved by the Bioethics Committee (“Comitato Etico Interaziendale”) of the S.S. Antonio e Biagio Hospital, Alessandria, Italy.

The cells were grown in Ham's F12 nutrient mixture medium, supplemented with 10% v/v fetal bovine serum (FBS), 1% v/v penicillin-streptomycin, 1% v/v L-glutamine, and were maintained in a humidified atmosphere at 37°C and 5% CO_2_.

### Cell viability

100,000 cells were seeded in 96-well plates, treated for 48 h with chemotherapeutic agents at scalar concentrations (from 1 pmol/L to 1 mmol/L), then stained with neutral red solution as described [44]. The IC50, i.e. the concentration of each drug that decreased the cell viability by 50%, was calculated with the CompuSyn software (http://www.combosyn.com). The Rf was calculated by dividing the IC50 of each drug in HMM cells for the IC50 of each drug in HMC. The synergistic, additive or antagonistic effect of the different concentrations of chemotherapeutic drugs in the presence of 1 μmol/L zoledronic acid was calculated with the CI equation of Chou-Talalay [45], using the CompuSyn software.

### Mevalonate pathway activity

The cells were labeled with 1 μCi/mL [^3^H]acetate (3600 mCi/mmol; Amersham Bioscience, Piscataway, NJ). The synthesis of radiolabeled cholesterol and FPP was measured as described [21].

### Ras activation assay

The GTP-bound Ras fraction, taken as an index of active G-proteins [46], was measured as reported [21].

### Western blotting

The cells were lysed in MLB buffer (125 mmol/L Tris-HCl, 750 mmol/L NaCl, 1% v/v NP40, 10% v/v glycerol, 50 mmol/L MgCl_2_, 5 mmol/L EDTA, 25 mmol/L NaF, 1 mmol/L NaVO_4_, 10 μg/mL leupeptin, 10 μg/mL pepstatin, 10 μg/mL aprotinin, 1 mmol/L phenylmethylsulfonyl fluoride, pH 7.5), sonicated and centrifuged at 13,000 x g for 10 min at 4°C. 20 μg of proteins from cell lysates were subjected to Western blotting and probed with the following antibodies: phospho(Thr202/Tyr204, Thr185/Tyr187)-ERK1/2 (Millipore, Billerica, MA); ERK 1/2 (Millipore); HIF-1α (BD Bioscience, San Jose, CA); Pgp/ABCB1 (Santa Cruz Biotechnology Inc., Santa Cruz, CA); MRP1/ABCC1 (Abcam, Cambridge, UK); MRP2/ABCC2 (Abcam); MRP3/ABCC3 (Santa Cruz Biotechnology Inc.); MRP4/ABCC4 (Abcam); MRP5/ABCC5 (Santa Cruz Biotechnology Inc.); BCRP/ABCG2 (Santa Cruz Biotechnology Inc.); IDO (Abcam); phospho(Tyr701)-STAT1 (Cell Signaling Technology, Danvers, MA); phospho(Ser727)-STAT1 (Millipore); STAT1 (Thermo Scientific, Rockford, IL); phospho(Tyr705)-STAT3 (Cell Signaling Technology); phospho(Ser727)-STAT3 (Cell Signaling Technology); STAT3 (Thermo Scientific); β-tubulin (Santa Cruz Biotechnology Inc.). The proteins were detected by enhanced chemiluminescence (Bio-Rad Laboratories). HIF-1α phosphorylation and nuclear translocation were measured as reported previously [21], using an anti-TATA-box binding protein (TBP) antibody (Santa Cruz Biotechnology Inc.) as a control of equal nuclear protein loading. To exclude any cytosolic contamination of nuclear extracts, we verified that β-tubulin was undetectable in nuclear samples (not shown).

### ERK, HIF-1 and DHFR activity

The ERK activity in whole cell lysates and the HIF-1 activity in nuclear extracts were measured with the MAP kinase (ERK1/2) Activity Assay kit (Millipore) and with the TransAM™ HIF-1 Transcription Factor Assay Kit (Active Motif, La Hulpe, Belgium), following the manufacturer's instructions. The activity of DHFR, a target enzyme of pemetrexed [47], was measured in whole cell lysates using the Dihydrofolate Reductase Assay kit (Sigma Chemical Co.), following the manufacturer's instructions. The data were expressed as units/mg proteins.

### Chromatin immunoprecipitation (ChIP)

ChIP experiments to measure the binding of HIF-1α to the hypoxia responsive element of the promoter of *MDR1* were performed as previously reported [48].

### Comet assay

The genotoxic damages, taken as indexes of cisplatin activity, were evaluated by the Single Cell Gel Electrophoresis assay (Comet assay), as reported previously [49]. Images were quantified by the CometScore software (TriTek Corp., Sumerduck, VA).

### Cell cycle analysis

The cell cycle distribution was analyzed as a parameter of gemcitabine efficacy [50]. Cells were washed twice with fresh PBS, incubated in 0.5 mL ice-cold ethanol 70% v/v for 15 min, then centrifuged at 1,200 x g for 5 min at 4°C and rinsed with 0.3 mL of citrate buffer (50 mmol/L Na_2_HPO_4_, 25 mmol/L sodium citrate, 1% v/v Triton X-100), containing 10 μg/mL propidium iodide and 1 mg/mL RNAse (from bovine pancreas). After a 15 min incubation in the dark, the intracellular fluorescence was detected by a FACSCalibur flow cytometer (Becton Dickinson). For each analysis, 10,000 events were collected and analyzed by the Cell QuestPro software (Becton Dickinson).

### IDO activity

The IDO activity was determined as previously characterized [51].

### Quantitative real time-PCR (qRT-PCR)

Total RNA was reverse-transcribed using the iScriptTM cDNA Synthesis Kit (Bio-Rad Laboratories). The qRT-PCR was performed with the iTaqTM Universal SYBR® Green Supermix (Bio-Rad Laboratories). The primer sequences were designed with the qPrimerDepot software (http://primerdepot.nci.nih.gov/). The relative quantification was performed by comparing each PCR product with the housekeeping PCR product (β-actin), using the Bio-Rad Software Gene Expression Quantitation (Bio-Rad Laboratories).

### Immunological assays

1 × 10^6^/mL human peripheral blood mononuclear cells (PBMC), isolated from buffy coats of healthy donors (kindly provided by Blood Bank, Città della Salute e della Scienza di Torino Hospital, Torino, Italy) by centrifugation on Ficoll-Hypaque density gradient, were treated with anti-CD3 (OKT3, BioLegend, San Diego, CA) and anti-CD28 (BioLegend) antibodies, to induce the specific proliferation of T-lymphocytes. Then, cells were co-cultured for 72 h with HMC or HMM cells, previously irradiated with 30 Gy for 15 min, at an effector/target cells ratio of 10:1. The expansion of T-lymphocytes, the only PBMC population able to proliferate in these experimental conditions, was assessed by adding 1 μCi of [^3^H]thymidine (PerkinElmer, Waltham, MA) 18 h before the end of the co-cultures, then harvesting the plates and counting the radioactivity. To analyze the lymphocyte phenotype after the incubation with tumor cells, the cells were harvested, washed and re-suspended in PBS containing 5% v/v FBS. A 3- and 4-color flow cytometry analysis was performed with the appropriate combinations of fluorescein isothiocyanate-, r-phycoerythrin-, Tricolor-, Peridinin Chlorophyll Protein Complex- or allophycocyanin-conjugated antibodies against CD3, CD4, CD8, CD25 (all from Miltenyi Biotech, Bergisch Gladbach, Germany) and CD127 (BioLegend). Isotype controls were run for each sample. The samples were read with a FACSCalibur flow cytometer equipped with Cell QuestPro software (Becton Dickinson). The use of PBMC from healthy donors and the experimental protocols were approved by the Bioethics Committee (“Comitato Etico Interaziendale”) of the Città della Salute e della Scienza di Torino Hospital, Torino, Italy.

### Cell silencing

For transient silencing of STAT1 and STAT3, 200,000 cells were transfected with 400 nmol/L of 19–25 nucleotide non-targeting scrambled siRNAs, with a STAT1- or STAT3-specific siRNAs pool (Santa Cruz Biotechnology Inc.). To produce HMM clones stably and inducibly silenced for Ras, 50,000 cells were transduced with 2.5 μg TetOn pTRIPZ vector containing a specific shRNA for *RAS* (Thermo Scientific), then selected in medium containing 1 μg/mL puromycin. *RAS* shRNA was induced by adding 1 μg/mL doxycycline for 24 h. To verify the silencing efficacy, the cells were lysed and checked for the expression of STAT1, STAT3 or Ras by Western blotting, as described above.

### Statistical analysis

All data in text and figures are provided as means ± SD. The results were analyzed by a one-way analysis of variance assay (ANOVA). *p* < 0.05 was considered significant.

## SUPPLEMENTARY FIGURES AND TABLES


