# Supplementary material for: Zoledronic acid overcomes chemoresistance and immunosuppression of malignant mesothelioma
Source: Oncotarget. 2014 Dec 31;6(2):1128–42. doi: 10.18632/oncotarget.2731 (PMC4359222; doi:10.18632/oncotarget.2731)
Supplement: Supplementary file 1 [file oncotarget-06-1128-s001.pdf]

SUPPLEMENTARY FIGURES AND TABLES

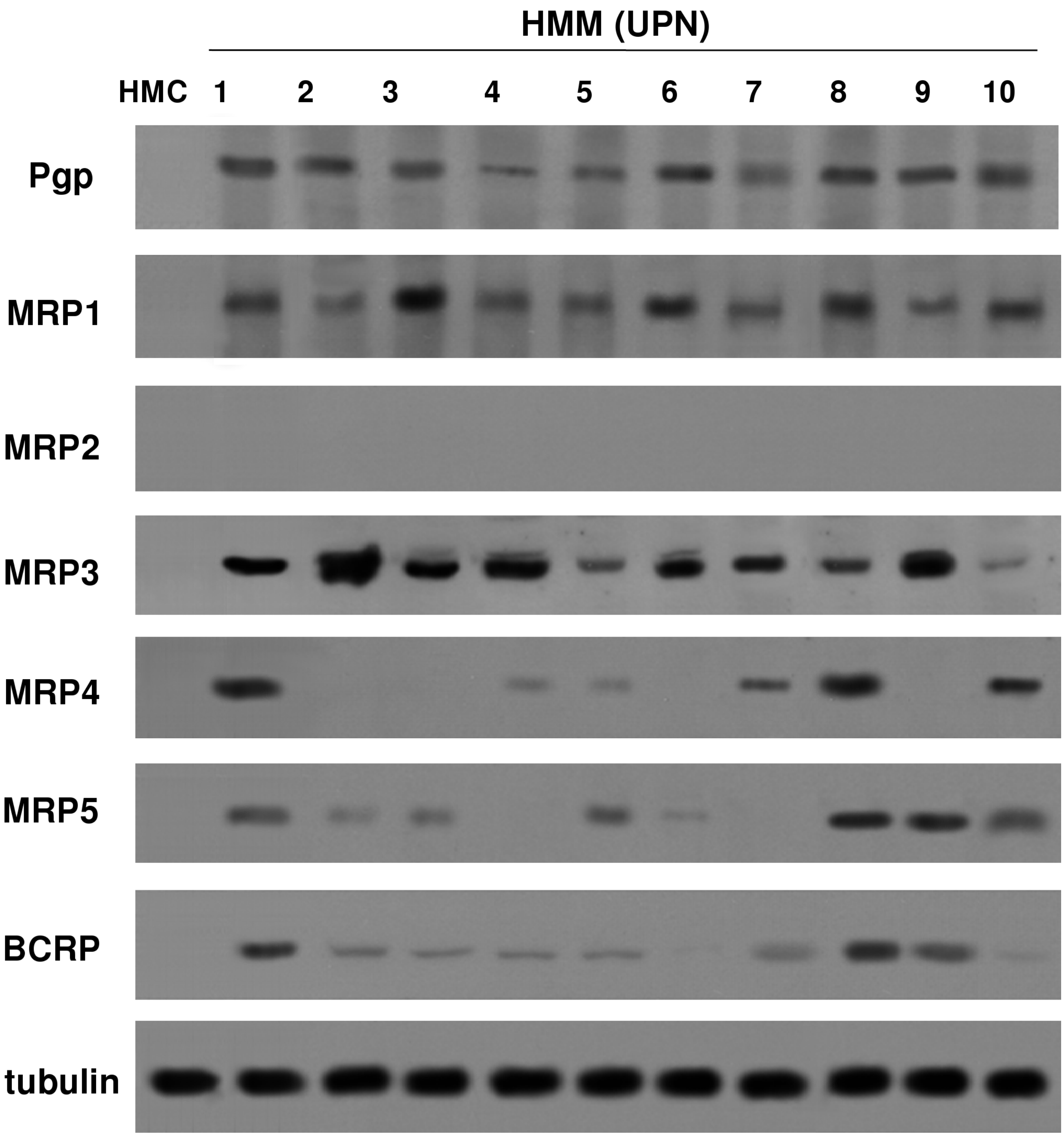

Supplementary Figure 1: Expression of ABC transporters in mesothelial and mesothelioma cells. HMC and HMM cells (UPN, unknown patient number) were lysed and subjected to the Western blot analysis for Pgp, MRP1, MRP2, MRP3, MRP4, MRP5, BCRP. The β-tubulin expression was used as control of equal protein loading. The figure is representative of 3 experiments with similar results.

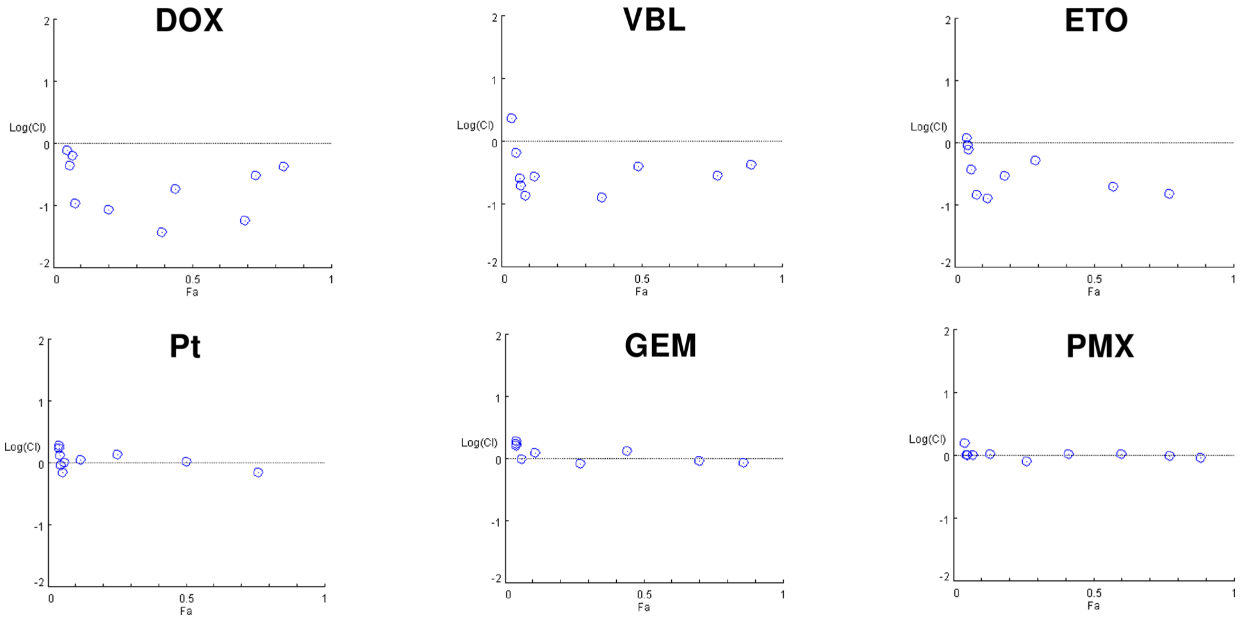

Supplementary Figure 2: Synergistic, additive and antagonistic effects of the association of zoledronic acid and chemotherapeutic drugs in mesothelioma cells. HMM cells were incubated for 48 h with 1 μmol/L zoledronic acid plus chemotherapeutic drugs (doxorubicin, DOX; vinblastine, VBL; etoposide, ETO; cisplatin, Pt; gemcitabine, GEM; pemetrexed, PMX) in a range of concentrations from 1 pmol/L to 1 mmol/L. The Fa-CI plot (Chou-Talalay's plot) was built by plotting the effect level (Fa, where 1 = 100% effect) versus the logarithm of the combination index (CI) for each combination of chemotherapeutic drugs and zoledronic acid. Each point represents the mean CI obtained in all HMM samples; the corresponding numerical values are reported in the Supplementary Table 2. Points below the horizontal line: synergism; points on the horizontal line: additive effect; points above the horizontal line: antagonism.

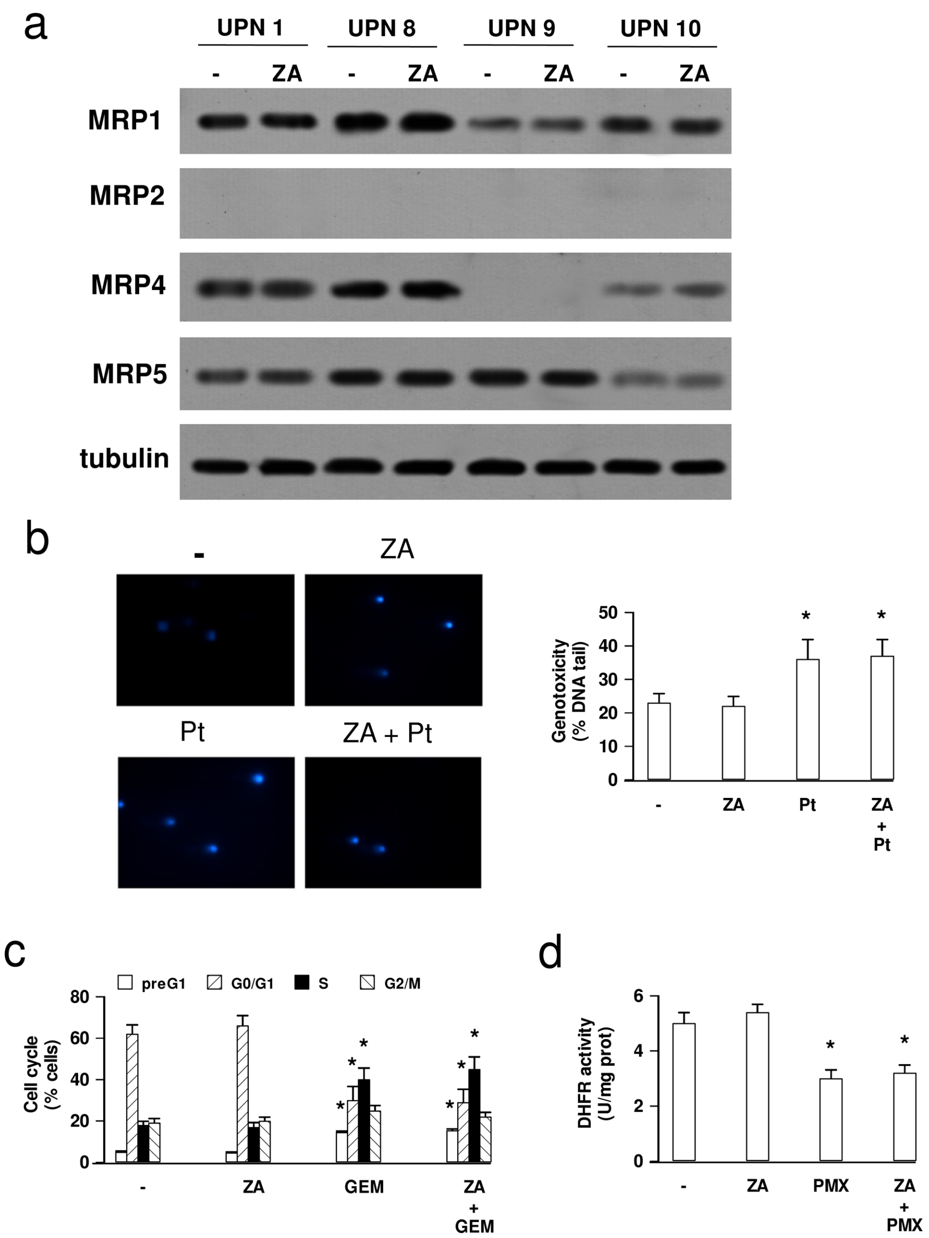

Supplementary Figure 3: Effect of zoledronic acid on ABC transporters and intracellular targets of cisplatin, gemcitabine and pemetrexed in mesothelioma cells. **(a)** HMM cells (UPN: unknown patient number) were incubated for 48 h in fresh medium (-) or with 1 µmol/L zoledronic acid (ZA), then lysed and subjected to the Western blot analysis for MRP1, MRP2, MRP4, MRP5. The β-tubulin expression was used as control of equal protein loading. The figure is representative of 3 experiments with similar results. **(c)** HMM cells were incubated for 48 h in fresh medium (-), in medium containing 1 µmol/L ZA or 50 µmol/L cisplatin (i.e. the mean IC50 for cisplatin; Pt), alone or in combination, then subjected to the Comet assay. Left panel: representative microphotographs of DNA comets in each experimental condition. The photographs are representative of independent experiments on the HMM samples of panel a. Bars = 10 µm. Right panel: quantitative analysis of the fluorescence microscope microphotographs. 100 tails for each experimental condition were analyzed. Vs untreated (-) cells: $^*p < 0.05$. HMM cells incubated for 1 h with 100 µmol/L H_2O_2, chosen as inducer of DNA damage, had a percentage of DNA tail of 64 ± 13% ($p < 0.01$ vs untreated cells) **(c)** HMM cells were grown for 48 h in fresh medium (-), in medium containing 1 µmol/L ZA or 30 µmol/L gemcitabine (i.e. the mean IC50 for gemcitabine; GEM), alone or in combination. The cell cycle distribution was measured by flow cytometry. Data are presented as means ± SD ($n = 3$). Vs untreated (-) cells: $^*p < 0.005$. **(d)** HMM cells were incubated for 48 h in fresh medium (-), in medium containing 1 µmol/L ZA or 6 µmol/L pemetrexed (i.e. the mean IC50 for pemetrexed; PMX), alone or in combination, then lysed and analyzed for the activity of dihydrofolate reductase (DHFR). Data are presented as means ± SD ($n = 3$). Vs untreated (-) cells: $^*p < 0.01$. HMM cells incubated for 48 h with 100 µmol/L methotrexate, chosen as positive control of DHFR inhibition, had an activity of 0.96 ± 0.13 U/mg proteins ($n = 3$; $p < 0.001$ vs untreated cells).

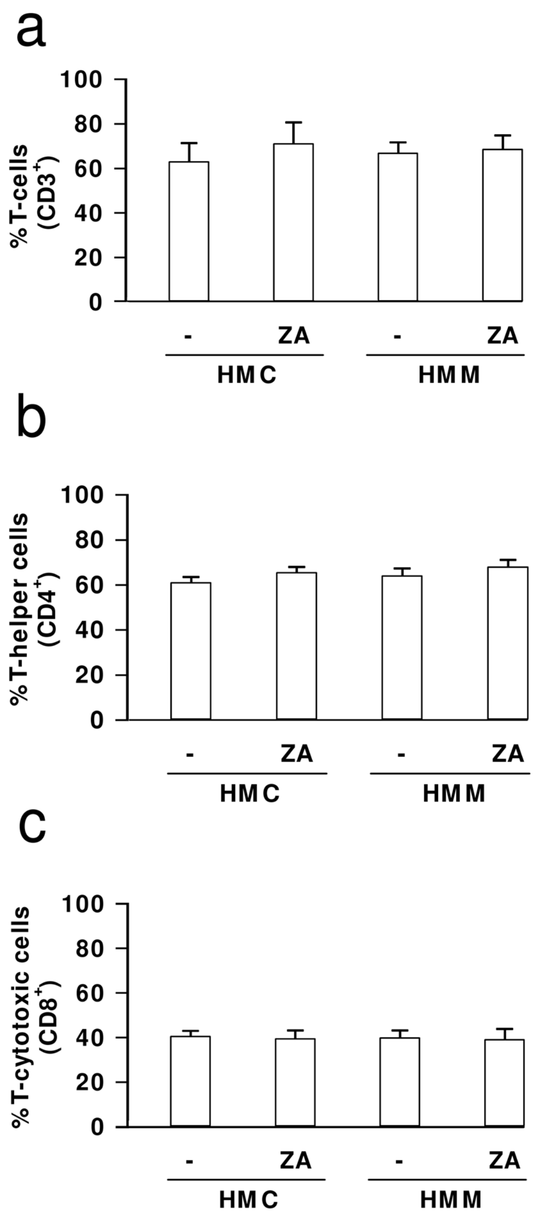

Supplementary Figure 4: Effects of zoledronic acid on $CD3^+$ T-cells, $CD4^+$ T-helper cells, $CD8^+$ T-cytotoxic cells expansion induced by mesothelial and mesothelioma cells. The percentage of $CD3^+$ T-cells (panel **a**), $CD4^+$ T-helper cells (panel **b**), $CD8^+$ T-cytotoxic cells (panel **c**) collected from PBMC, stimulated with anti-CD3 and anti-CD28 antibodies, and co-incubated for 72 h with HMC and HMM cells, grown in fresh medium (-) or in medium containing 1 µmol/L zoledronic acid (ZA), was measured by flow cytometry. Data are presented as means ± SD ($n = 3$).

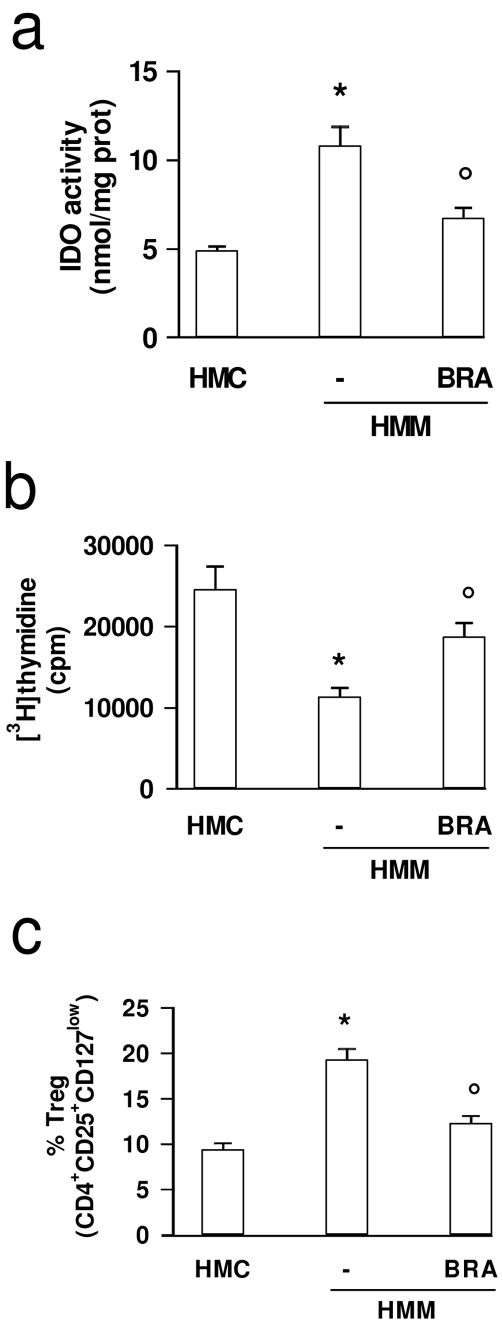

Supplementary Figure 5: Effects of an IDO inhibitor on the expansion of T-lymphocytes and Tregs induced by mesothelioma cells. (a) HMM cells were incubated in fresh medium (-) or in the presence of the IDO inhibitor 5-Br-brassinin (100 µmol/L, BRA) for 72 h. HMC were used as internal control. The kynurenine level in the cell culture supernatant, taken as an index of IDO enzymatic activity, was measured fluorimetrically. Data are presented as means ± SD ($n = 3$). HMM cells vs HMC: $^*p < 0.001$; BRA-treated HMM cells vs untreated (-) cells: $^\circ p < 0.005$. **(b)** The proliferation of activated T-lymphocytes collected from PBMC after a 72 h co-incubation with HMC or HMM cells, grown in the absence (-) or in the presence of BRA, was measured with the $[^3H]$thymidine assay. As positive control of proliferation, the PBMC were treated with the anti-CD3 and anti-CD28 antibodies, in the absence of cells; as negative control, the PBMC were grown in RPMI-1640 medium, in the absence of anti-CD3 and anti-CD28 antibodies, and of cells. In the presence of the anti-CD3 and anti-CD28 stimulatory antibodies without cells, the $[^3H]$thymidine incorporation was 30,918 ± 1,781 cpm; in the presence of RPMI-1640 medium alone, the $[^3H]$thymidine incorporation was 4,025 ± 612 cpm. Data are presented as means ± SD ($n = 2$). HMM cells vs HMC: $^*p < 0.01$; BRA-treated HMM cells vs untreated (-) cells: $^\circ p < 0.02$. **(c)** The percentage of Tregs ($CD4^+CD25^+CD127^{low}$) collected from PBMC, co-incubated as reported in **b**, was measured by flow cytometry. Data are presented as means ± SD ($n = 2$). HMM cells vs HMC: $^*p < 0.001$; BRA-treated HMM cells vs untreated (-) cells: $^\circ p < 0.001$.

Supplementary Table 1: Chemotherapeutic drugs used in the study and relative efflux transporters

Drug	Transporter
doxorubicin	Pgp, MRP1, MRP2, MRP3, BCRP
vinblastine	Pgp, MRP1, MRP2
etoposide	Pgp, MRP1, MRP2, MRP3
cisplatin	MRP1, MRP2, MRP4
gemcitabine	MRP5
pemetrexed	MRP5
mitoxantrone	BCRP, Pgp, MRP1

Supplementary Table 2: Combination index (CI) of zoledronic acid and chemotherapeutic drugs in HMM cells

ZA	drug	DOX + ZA	VBL + ZA	ETO + ZA	Pt + ZA	GEM + ZA	PMX + ZA
(µmol/L)	(µmol/L)	CI	CI	CI	CI	CI	CI
1	0.000001	0.11	0.42	1.19	1.92	1.93	1.58
1	0.00001	0.79	0.52	0.92	1.74	1.75	1.03
1	0.0001	0.44	0.19	0.78	1.31	1.59	1.01
1	0.001	0.64	0.09	0.37	0.93	1.73	1
1	0.01	0.08	0.14	0.15	0.69	0.98	1.06
1	0.1	0.04	0.28	0.13	1.01	1.24	0.81
1	1	**0.18**	**0.13**	0.31	1.35	0.83	**1.06**
1	10	**0.06**	**0.41**	**0.52**	**1.04**	**1.31**	**1.07**
1	100	0.31	0.28	**0.21**	**1.11**	**0.92**	0.97
1	1000	0.46	0.43	0.14	0.71	0.86	0.93

ZA: zoledronic acid; DOX: doxorubicin; VBL: vinblastine; ETO: etoposide; Pt: cisplatin; GEM: gemcitabine; PMX: pemetrexed.

CI was calculated in human malignant mesothelioma HMM cells incubated for 48 h with 1 µmol/L ZA plus chemotherapeutic drugs in a range of concentrations from 1 pmol/L to 1 mmol/L, using the Compusyn software.

CI < 0.10: very strong synergism; 0.10 < CI < 0.30: strong synergism; 0.30 < CI < 0.70: synergism; 0.70 < CI < 0.85: moderate synergism; 0.85 < CI <0.90: slight synergism; 0.90 < CI <1.10: additive effect; 1.10 < CI < 1.20: slight antagonism; 1.20 < CI < 1.45: moderate antagonism; 1.45 < CI < 3.30: antagonism; 3.30 < CI < 10: strong antagonism; CI > 10: very strong antagonism. Bold values highlight the CI falling in the range of the IC50 of each drug in the presence of ZA.
